# Dinuclear Macrocyclic Bis(iminopyridyl) Co- and Fe-Based Catalysts for Ethylene Oligomerization

**DOI:** 10.3390/ma18092123

**Published:** 2025-05-05

**Authors:** Mostafa Khoshsefat, Yanping Ma, Wen-Hua Sun

**Affiliations:** 1 Graduate School of Advanced Science and Technology, Japan Advanced Institute of Science and Technology, 1-1 Asahidai, Nomi 923-1292, Ishikawa, Japan; 2 Key Laboratory of Engineering Plastics and Beijing National Laboratory for Molecular Science, Institute of Chemistry Chinese Academy of Sciences, Beijing 100190, China; myanping@iccas.ac.cn; 3 State Key Laboratory for Oxo Synthesis and Selective Oxidation, Lanzhou Institute of Chemical Physics Chinese Academy of Sciences, Lanzhou 730000, China

**Keywords:** dinuclear catalyst, macrocyclic ligand, iron, cobalt, oligomer, cooperative effect

## Abstract

Recent advances in designing multinuclear late transition metal catalysts for the oligo-/polymerization of olefins emphasize the great interest and promising approaches in the preparation and application of these catalytic systems. Accordingly, in this study, two dinuclear macrocyclic bis(iminopyridine) Fe- and Co-based complexes (FC and CC) were prepared at moderate yields through a one-pot template reaction. Upon activation by MMAO, not only did the catalysts show reasonable activities for the oligomerization of ethylene but also showed high selectivity for the production of tetramers (α-C_8_). With respect to the catalyst structure, FC demonstrated higher catalyst activity (9.45 g mol^−1^ Fe h^−1^ × 10^5^ vs. 8.75 × 10^5^ g mol^−1^ Co h^−1^) along with higher selectivity for α-C_8_ production compared to CC (96.6 vs. 96.1%). Both catalysts had thermal stability up to 70 °C, with FC being much more active and stable than CC under identical conditions. On the other hand, polymerization parameters had an influence on the catalyst performance and oligomer distribution. Moreover, molecular calculations were employed for geometry optimization and structural determination, which was consistent with the experimental results.

## 1. Introduction

The production of oligomers has always been of interest to researchers and industrial experts, especially in the petrochemicals industry [1]. Due to the large market demand and value-added potential of linear α-olefins (LAOs), they have become one of the most needed products. Since 2003, chromium-based catalysts have been widely developed and commercialized for the selective oligomerization—particularly trimerization—of ethylene, owing to their excellent performance in producing LAOs [2,3,4,5,6,7,8]. On the other hand, over the past few decades, a variety of alternative catalyst systems have been introduced for the oligomerization of ethylene into dimers, trimers, and higher LAOs [9,10]. Among these, late transition metal (LTM) catalysts have emerged as particularly promising candidates [10,11,12,13,14]. Most monometallic LTM examples are based on iron or cobalt coordinated to [N,N,N] or [N,N,O] pincer-type ligands, as well as nickel complexes featuring [N,N] pincer ligands. In addition to the mononuclear systems, multinuclear catalysts have also gained significant attention due to their potential for enhanced reactivity and selectivity [15]. The structural diversity of LTM catalysts allows for synergistic and cooperative effects, which can positively influence both the catalytic performance and the properties of the resulting products [16,17,18,19,20].

**Scheme 1:**
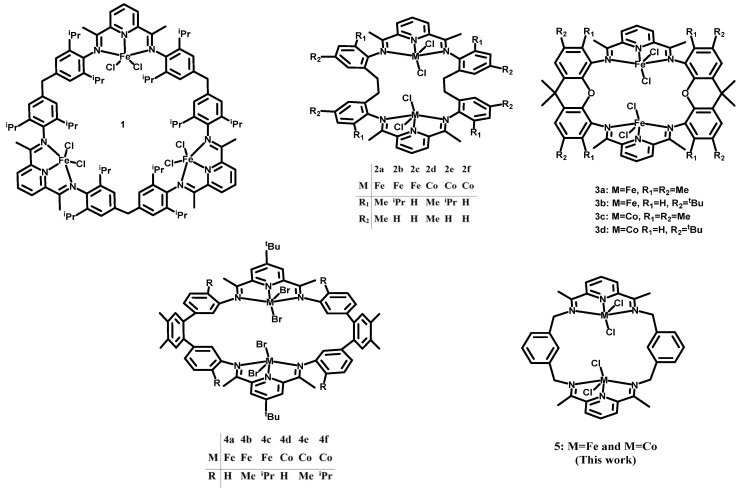
Dinuclear macrocyclic Fe- and Co-based structures used for ethylene polymerization. The abbreviations used are as follows: Me = methyl, iPr = isopropyl, and tBu = tert-butyl.

Among the different building blocks of ligands already employed to make these structures, macrocyclic scaffolds are noteworthy structures (Scheme 1; **1**–**4**) [21,22,23,24,25,26]. For instance, the trinuclear macrocyclic Fe-based catalyst **1** reported by Liu et al. exhibited high activity up to 4.3 × 10^6^ g (PE) mol^−1^ (Fe) h^−1^ in the presence of MMAO (PE: polyethylene and MMAO: modified-methylaluminoxane) [23]. In terms of the properties of the PE produced using **1**, it had much higher *M_w_* and melting points than those made using its mononuclear analogue. In addition, **1** demonstrated higher stability and longer lifetime compared to the mononuclear analogue. The authors, using mechanics calculation, explained that these observations are due to the nature of the catalyst, which repels the deactivation mechanism and regulates the chain transfer reactions.

A series of dinuclear Co- and Fe-based catalysts (**2a**–**2f**) were synthesized by Takeuchi et al. [24]. These double-decker structures were highly thermally stable (up to 120 °C) and active (4.9 × 10^6^ g (PE) mol^−1^ (Co) h^−1^ and 2.0 × 10^6^ g (PE) mol^−1^ (Fe) h^−1^). The Co-based ones resulted in a PE with a higher *M_w_* than their corresponding mononuclears. The results were attributed to the cooperative effect, where an agostic interaction between the polymeryl species linked to a center and a second metal center may repel the chain transfer and deactivation processes. They also used similar backbones that were linked by 4,5-xanthene moieties to prepare **3a**–**3d** [21]. In comparison to the corresponding Fe and Co catalysts, almost similar activities were observed; however, **3a** was capable to produce oligomers containing ethyl and/or propyl branches, implying the cooperative interactions. In another report by the Jordan group, macrocyclic structures connected by *o*-terphenyl segments (**4a**–**4f**) were prepared [25]. Among these structures, the thermally stable catalyst **4c**, having *iso*-propyl groups on the *o*-positions, displayed the highest catalyst activity up to 7.8 × 10^6^ g (PE) mol^−1^ (Fe) h^−1^ (upon activation by triisobutylaluminum (TiBA)). Besides, **4b** and **4c** resulted in the production of PE with a broad range of molecular weights (*M_w_* = 4.5 × 10^3^−2.8 × 10^5^ g mol^−1^), while **4a** and **4d** bearing H atoms on the *o*-position of terphenyl groups produced oligomers (1-butene as the major product).

Herein, we synthesized two dinuclear macrocyclic Fe- and Co-based complexes (Scheme 1; **5**). The design of the structures was based on the following factors: (a) The novelty of the structures for oligomerization—specifically, using simple linkers without alkyl groups on the spacer to avoid blocking the *ortho*-positions of the active sites; (b) Positioning of the active centers at an optimal distance to maximize the bimetallic effects; (c) Accessibility and feasibility of the synthetic route required to obtain the desired structures. With regard to the last factor, *m*-xylylene was found to be the most suitable spacer for successfully obtaining the final complexes. These catalysts, then, were employed for ethylene oligomerization in the presence of modified-methylaluminoxane (MMAO) as cocatalysts. The effect of polymerization parameters (time, temperature, [Al]/[M] molar ratio, and monomer pressure) was studied to have better control on the catalyst behavior and oligomer distribution.

## 2. Materials and Methods

### 2.1. General Procedure and Materials

All manipulations of air-/water-sensitive compounds were conducted under Ar/N2 atmosphere using the standard Schlenk technique. All the solvents were purified prior to use. 2,6-diacetylpyridine, *m*-xylylene diamine, cobalt(II) chloride hexahydrate, and iron (II) chloride tetrahydrate were purchased from Sigma Aldrich Chemicals (Steinheim, Germany). Toluene (purity 99.9%) was purified over sodium wire/benzophenone and used as polymerization media. Ethylene (purity 99.99%) was provided by Bandar Imam Petrochemical Company (BIPC, Iran). Modified-Methylaluminoxane (MMAO) (7 wt% in Toluene) was supplied by Sigma Aldrich Chemicals (Steinheim, Germany).

### 2.2. Synthesis of CC

A suspension of 2,6-diacetylpyridine (0.19 g, 1.0 mmol), *m*-xylylene diamine (0.14 g, 1.0 mmol), and CoCl_2_·6H_2_O (0.26 g, 1.1 mmol) in glacial acetic acid (15 mL) was stirred and heated to reflux for 12 h. Upon cooling to room temperature, an excess of diethyl ether was added to precipitate the crude product that was collected before being redissolved in methanol. The methanol solution was concentrated, and the product was precipitated again with diethyl ether. The solid was dried under reduced pressure and obtained as a brown powder (65%). Data for CC: ^1^H NMR (300 MHz, THF-d_8_, TMS); δ 7.93 (2H), 7.25 (12H), 4.76, 4.61 (8H), and 2.28 (12H). FT-IR (KBr, cm^−1^): 1695 cm^−1^ (-C=N-) and 803 cm^−1^ (Co-Cl). Anal. calculated for C_34_H_34_Cl_4_Co_2_N_6_: C, 51.93; H, 4.36; and N, 10.69. Found: C, 51.20; H, 3.71; and N, 9.87%. MS (MALDI-TOF): *m/z* 787.79 [M + H]^+^.

### 2.3. Synthesis of FC

Using a similar procedure and molar ratios to that described for the synthesis of CC, except using FeCl_2_·4H_2_O instead of CoCl_2_·6H_2_O, FC was isolated as a dark-blue powder (71%). Data for FC: ^1^H NMR (300 MHz, THF-d_8_, TMS); δ 7.78 (2H), 7.31 (12H), 4.36, 4.30 (8H), and 1.95 (12H). FT-IR (KBr, cm^−1^): 1690 cm^−1^ (-C=N-), 793 cm^−1^ (Fe-Cl-Fe), and 777 cm^−1^ (Fe-Cl). Anal. calculated for C_34_H_34_Cl_3_Fe_2_N_6_: C, 54.84; H, 4.60; and N, 11.28. Found: C, 54.69; H, 4.51; and N, 11.08%. MS (MALDI-TOF): m/z 745.85 [M + H]^+^.

### 2.4. Ethylene Oligomerization

Ethylene oligomerizations were conducted in a 200 mL stainless steel Buchi reactor using toluene as the solvent. Reactor was purged with nitrogen at 90 °C for 2 h prior to each reaction. Dried toluene was introduced under nitrogen atmosphere, and MMAO was used as a cocatalyst. Reactor was saturated with ethylene to the desired total pressure (1, 2, and 4 bar), and the reaction proceeded for 20 min by mixing at 800 rpm. To better study the reaction coordinates, stirrer was stopped while injecting the catalyst, and then stirring and plotting were started concurrently. Ethylene consumption was compensated using a mass flow meter to keep the pressure constant. Finally, the reactor contents were evacuated, and a small amount of the reaction solution was collected for GC–MS analysis and NMR studies.

### 2.5. Characterization

FT-IR spectrums were obtained using Thermo Nicolet AVATAR 370 spectrometer. Elemental analysis was performed on a Thermo Finnigan Flash 1112EA microanalyzer. GC–MS analyses were performed on Agilent 6890 series GC, with Agilent 5973 Network MS detector system equipped with a 30 m (0.25 mm i.d., 0.25 μm film thickness) VARIAN, VF-1ms column. Mass analysis on the complexes was performed using Bruker Daltonics flex Analysis (ultraflexTOF/TOF), and NMR spectrum was recorded from Bruker DMX 300 spectrometer.

## 3. Results and Discussion

### 3.1. Synthesis and Characteristics of Complexes

**Scheme 2:**
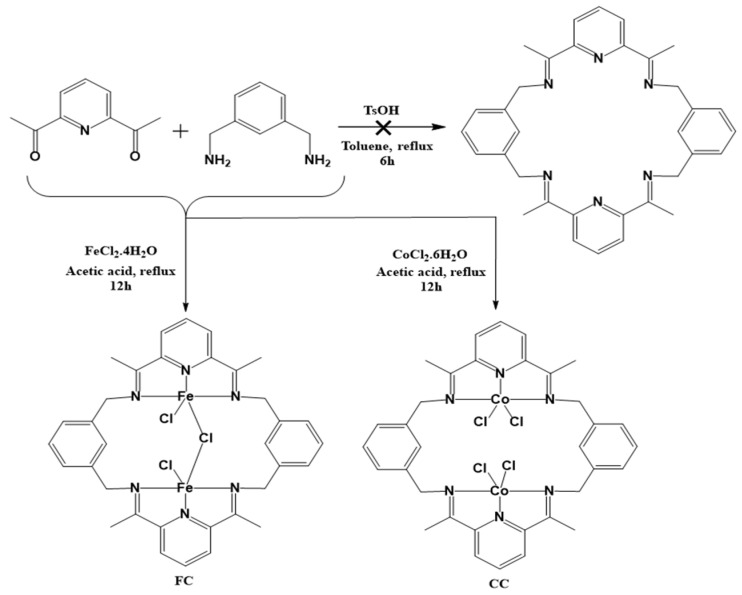
Synthesis route for dinuclear macrocyclic complexes (FC and CC).

The reaction between 2,6-diacetyl pyridine and *m*-xylylene diamine leads to a polymeric ligand and does not give the macrocyclic structure due to geometrical encumbrance. However, in order to overcome this obstacle, we used the one-pot synthesis route to prepare the complexes (CC and FC), as depicted in Scheme 2. Previously, this method has been employed for the facile synthesis of mono- and dinuclear complexes [26,27]. After preparation of the complexes, they were subjected to basic characterization techniques, including ^1^H NMR, FT-IR, elemental analysis, and MALDI-TOF. It was found that there is a μ–Cl between the Fe metal atoms, while in the case of Co (i.e., CC), there is no bridge.

Although the attempts for growing crystals from the complexes were not successful, we tried to optimize the complexes to obtain the most stable structures. For this purpose, a series of DFT studies were carried out.

### 3.2. DFT Studies

**Figure 1:**
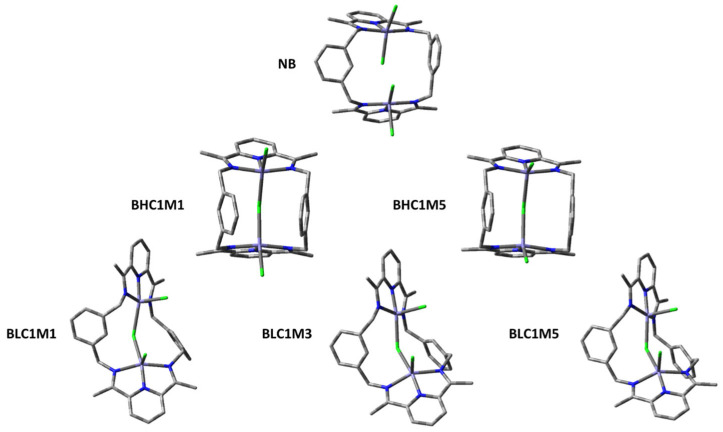
Optimized molecular structure of Fe-none-bridged (NB), bridged high constrained with charge 1 and multiplicity 1 (BHC1M1), bridged high constrained with charge 1 and multiplicity 5 (BHC1M5), bridged low constrained with charge 1 and multiplicity 1 (BLC1M1), bridged low constrained with charge 1 and multiplicity 3 (BLC1M3), and bridged low constrained with charge 1 and multiplicity 5 (BLC1M5).

**Figure 2:**
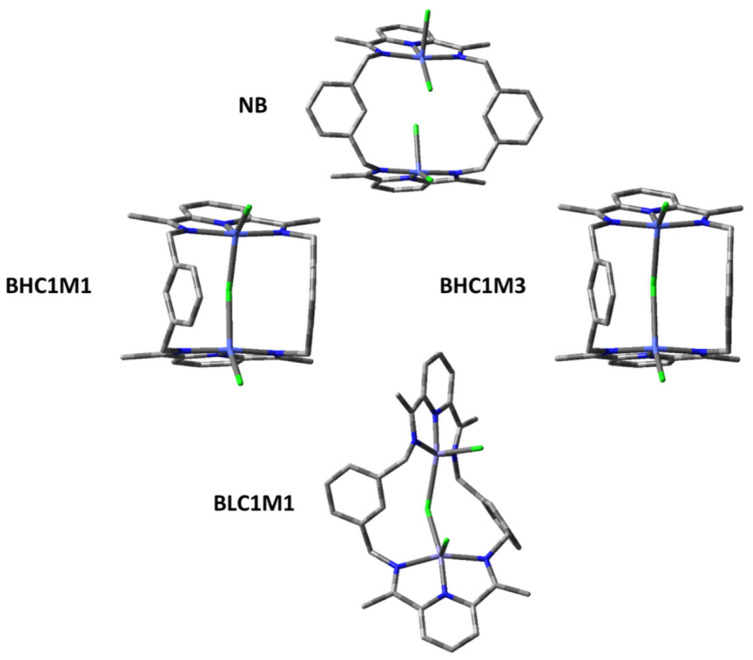
Optimized molecular structure of Co-none-bridged (NB), bridged high constrained with charge 1 and multiplicity 1 (BHC1M1), bridged high constrained with charge 1 and multiplicity 3 (BHC1M3), and bridged low constrained with charge 1 and multiplicity 1 (BLC1M1).

**Figure 3:**
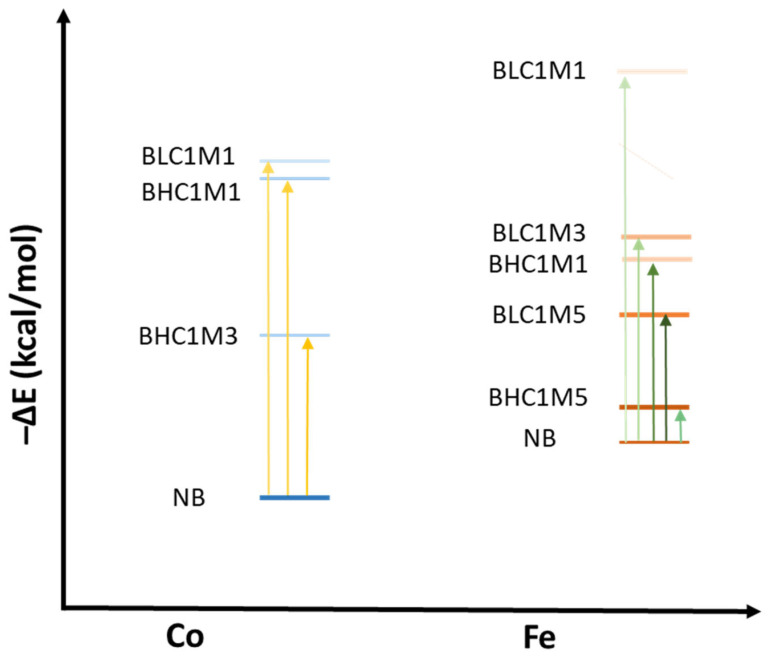
Comparison of the energies for the optimized structures. NBs were considered as the ground state.

According to the experimental analyses, it was observed that there is a difference between the FC and CC complexes, as the Fe metal centers are linked in FC through Cl-bridging. Both Co and Fe are transition metals that can form halide-bridged complexes due to their ability to exist in multiple oxidation states (II and III), which allows them to coordinate with halide ions [28,29,30,31,32,33,34]. In addition to this, their partially filled d-orbitals allow the metals to form coordination complexes with halides, where the halides (II) act as bridging ligands between metal centers. The ionic nature of halides and their ability to undergo ligand exchange make them effective at stabilizing these complexes. Additionally, electron delocalization and favorable geometries further enhance the stability of the metal–halide–metal linkages. Based on the reports, it can be observed that for similar structures, the formation of dinuclear Fe complexes bearing a chloride-bridged structure is more probable than Co complexes due to differences in their electronic structure, oxidation states, and metal–metal interactions [35,36,37,38]. Accordingly, Fe (II) with its smaller ionic radius and high charge density forms strong metal–metal bonds and is more likely to be bridged by chloride ions. In contrast, Co (II) and Co (III) have larger ionic radii and lower charge densities, making them less prone to such bonding and less able to form stable chloride-bridged complexes. Additionally, Fe’s coordination environment allows for chloride bridging, while Co prefers isolated, mononuclear structures. This combination of factors leads to the formation of chloride-bridged dinuclear complexes in Fe, but not in Co. However, in order to better study the molecular structures of the complexes, density functional theory (DFT) calculations at the b3lyp/6-31g level of the theory were applied for the Fe and Co complexes at different multiplicities (charge, C and multiplicity, M), geometries (low and high constrained, L and H), and halide-bridging (bridged and isolated), as provided in Figure 1, Figure 2 and Table 1. In addition, the energies for the optimized structures were obtained using ub3lyp/6-31g and GD3 in order to correct the empirical dispersion of aromatic rings, and the results are depicted in Figure 3, where the non-bridged (NB, C = 0 and M = 1) structure, with metals isolated and linked with two chloride atoms, was considered as a ground state; in contrast, in the chloride bridged (μ-Cl) structures (B_X_C_y_M_z_; x = L and H, y = 1 and z = 1, 3 and 5), the level of energies is the sum of the energy of each complex and an isolated Cl compared to the ground state (NB). According to Figure 1 and Figure 2, it can be seen that the different configurations are a result of the flexibility in the -CH_2_- of xylylene groups posing the structures in different directions, whether the metals are Cl-bridged or isolated. In the case of optimized structure for NBs, the xylylene bridges are aligned in CC, while they are perpendicular in the case of FC. Furthermore, the different configurations of backbones were aligned along the various angles between the spacers for the Cl-bridged structures.

In terms of the energies (Figure 3), it can be expected that NBs are the most stable structures, especially for CCs; however, the energy intervals between the NB and the most stable Cl-bridged structure for CC and FC are 142.5 kcal/mol and 8.5 kcal/mol, respectively; these can be considered as the representative structures based on the experimental results. Furthermore, the study of the molecular orbitals of the CC structures (Figure S9) showed that the electron density was primarily localized on the metal center and partially delocalized into the surrounding ligands in the case of HOMO, while in LUMO, it is more ligand-centered, particularly on the π* orbitals of a chelating ligand of CC. These orbitals for FC showed a difference in terms of more delocalization into ligand orbitals and metal–ligand π interaction compared to CC (HOMO). The LUMO of FC was slightly more diffuse or spread out than that of CC.

### 3.3. Ethylene Oligomerization Using FC and CC

**Figure 4:**
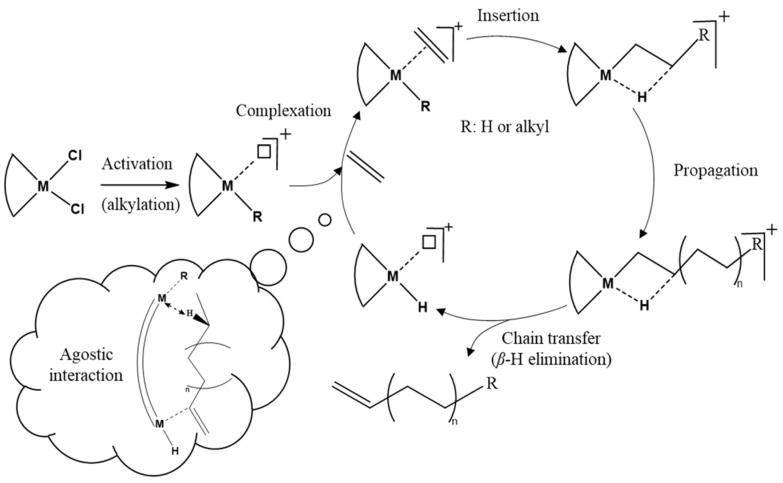
General mechanism of ethylene oligomerization catalyzed by mono- and dinuclear catalysts.

Upon activation with MMAO, both catalysts, CC and FC, exhibited promising activity toward ethylene oligomerization. A general mechanism of ethylene oligomerization catalyzed by mononuclear catalysts is provided in Figure 4. Furthermore, the plausible agostic interaction between the H of oligomer/-yl species and the second metal centers is depicted. This interaction was previously proposed for the cooperative metal–metal effect leading to higher performance of di- or multi-nuclear catalysts [15,16,20].

**Figure 5:**
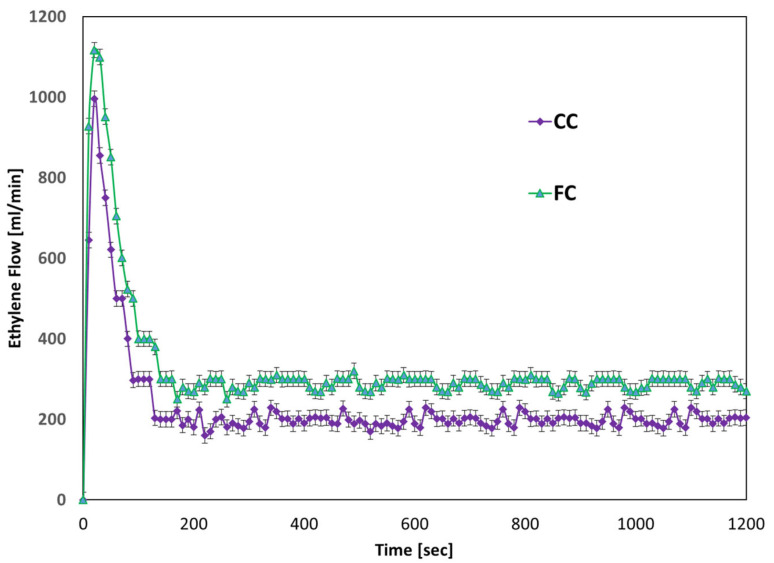
Normalized ethylene consumption profiles for dinuclear macrocyclic catalysts CC and FC (Table 1: entries 1 and 3).

To compare the kinetics of oligomerization by the catalysts, the curves of reaction time against ethylene flow consumption obtained by the mass flow controller (MFC) system are depicted in Figure 5. This kinetic profile clearly showed the higher activity of FC compared to CC [39]. Besides, not only was the ethylene uptake by FC more intense during the initial time of polymerization (up to 180 s), but also at longer times, the profile of FC stood at upper ethylene flow consumption. It can also be mentioned that the type of kinetic profile is build-up, excluding the first 3 min (180 s) of the consumption profile (as this period includes the pressurizing of the reactor and fast initial reactions after precatalyst injection); the highest activity can be observed even within the first stages of the reaction. Based on this, FC and CC exhibit similar profiles; however, the higher activity of FC may be attributed to the metal center and the configuration of the spacer. In CC, the aryl rings are aligned in the same plane, forming a fully open window. In contrast, in FC, the aryl rings are either perpendicular to each other (as in NB) or aligned in different planes (as in BHC1M5), as the interval energy between these structures is low (Figure 3), resulting in half-open or fully half-open window configurations. These structural differences may contribute to the higher performance and selectivity (as a cooperative effect) observed in FC compared to CC.

The activities of the catalysts and oligomer distributions were calculated using GC–MS analysis, and the results are tabulated in Table 1. The quantitative results were consistent on the ethylene uptake profiles, where FC displayed a higher activity for ethylene oligomerization than CC. Accordingly, the highest activity obtained for FC was 8.05 × 10^5^ g mol^−1^ Fe h^−1^, while for CC, it was 5.15 × 10^5^ g mol^−1^ Co h^−1^ under similar conditions.

### 3.4. Product Distribution and Microstructural Properties

**Figure 6:**
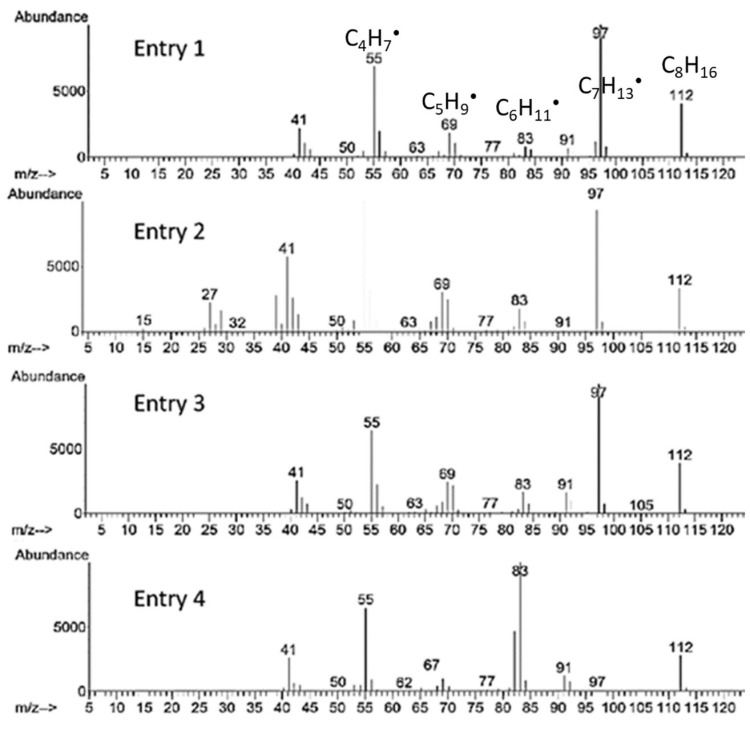
GC–MS spectra of the oligomers made by FC and CC (entries 1–4).

**Figure 7:**
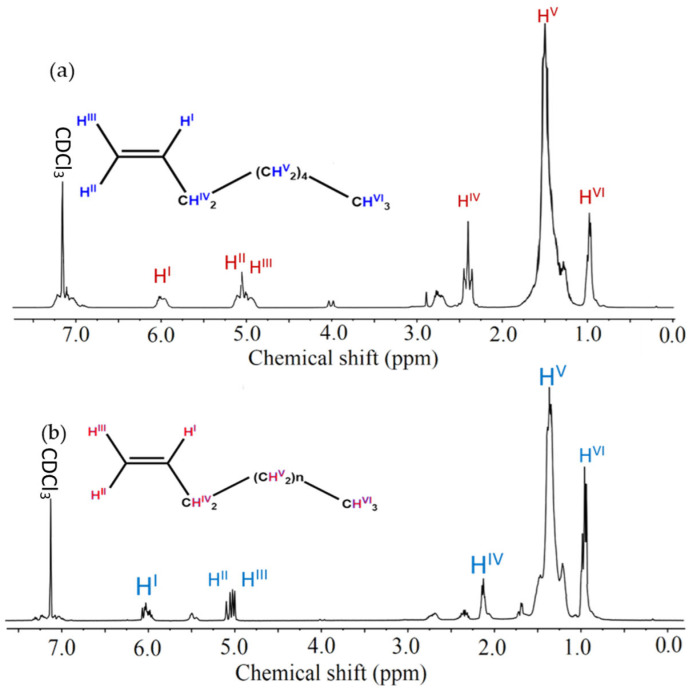
^1^H NMR spectra of the ethylene oligomers (in CDCl_3_) made by (**a**) CC (entry 4) and (**b**) FC (entry 2).

The most interesting aspect of the results was regarding the oligomer distributions and selectivity of the catalysts. High percentages of ethylene tetramers (C_8_) were generated by both the catalysts, CC and FC. The magnitude of selectivity for tetramerization by FC was 99.1%, where the ratio of α-C_8_ to the whole fraction of C_8_ was 96.4% (Table 1). On the other hand, CC was able to produce octane with lower selectivity up to 96.2%, with a higher ratio of unsaturated content to total alkane (α-C_8_/ƩC_n_). It was already discussed that the cobalt catalysts are inherently more selective for unsaturated content due to the electronic and steric properties of the metal center [40,41]. Although for both catalysts, selectivity slightly decreased as the monomer pressure was increased from 2 to 4 bar, α-C_8_ was still the major product. On the other side, the α-C_8_/ƩC_8_ ratio virtually increased, indicating that there is a higher rate of *β*–hydrogen elimination at upper monomer pressure. The GC–MS and ¹H NMR spectra of the oligomers produced by the catalysts are presented in Figure 6 and Figure 7. According to the chemical shift assignments, the structure of the majority of α-C_8_ oligomers was in line with the GC–MS analysis. The corresponding molecular formulas for the given ion or the mass of the fragments are provided in Figure 6 (C_8_H_16_; 1-octene, C_7_H_13_^•^; first major species after the fragmentation of 1-octene). Higher productivity and selectivity achieved by these catalysts strongly magnified the effect of dinuclearity on the catalyst behavior. To the best of our knowledge, these novel structures are the first class of dinuclear LTM catalysts having high selectivity for the tetramerization of ethylene. It has also been observed in some other dinuclear LTM catalysts developed by our group that a strong agostic interaction occurs in structures containing eight carbon atoms in the main chain with a second metal center, whether the structure originates from a 1-octene monomer or a linked polymer chain [20]. For these catalysts, the production of ultra-high molecular weight poly(1-octene) and hexyl-branched polyethylene provided supporting evidence for this explanation [18,20].

The ^1^H NMR analysis results of the oligomers made by CC and FC were completely in line with the GC–MS characterization data. As depicted in Figure 7, different hydrogens in the microstructure of oligomers have different chemical shifts. The hydrogens in the vinylic section of oligomers (H^I^, H^II^, and H^III^) were assigned to peaks in the ranges of 5.9–6.1 ppm and 4.9–5.2 ppm. For the rest of the hydrogen atoms (i.e., H^IV^, H^V^, and H^VI^), peaks appeared at the up-field areas (0.9–2.2 ppm), as they are shielded. All the peak assignments are depicted in Figure 7. The main difference between the ¹H NMR spectra of the oligomers produced by CC and FC lies in the appearance of minor peaks, which result in slightly broader signals due to the presence of shorter α-olefin fractions.

### 3.5. Effect of Polymerization Parameters

To better control and understand the impact of polymerization parameters on catalyst productivity and oligomer selectivity, reactions were carried out under varying conditions. The results of these experiments are summarized in Table 2. Both dinuclear macrocyclic catalysts demonstrated high thermal stability, maintaining ethylene polymerization activity at elevated temperatures up to 75 °C. However, higher reaction temperatures resulted in decreased selectivity for α-C_8_ and increased formation of shorter α-olefins, such as α-C_4_ and α-C_6_. This shift can be attributed to the increased kinetic energy of species in the reaction medium, which facilitates chain termination at elevated temperatures. A similar effect was observed with high cocatalyst concentrations, which enhanced the chain transfer reactions. Regarding the catalytic activity, higher cocatalyst concentrations led to lower productivity, likely due to catalyst deactivation [42]. The oligomerization of ethylene at high concentrations of monomer led to higher productivity for both catalysts, FC and CC (Table 1 and Table 2). However, it displayed a reverse effect on the selectivity of the catalysts, and the obtained oligomers had broader distributions. This may imply that higher pressure causes non-uniform chain propagation and transfer, thereby reducing the effective cooperative effect.

## 4. Conclusions

Two dinuclear macrocyclic bis(iminopyridine) Co- and Fe-based complexes (CC and FC) were synthesized in reasonable yields via a one-pot template reaction, an efficient and straightforward method. Their structures were confirmed through both experimental and computational analyses. These catalysts exhibited not only high activity in ethylene oligomerization but also high selectivity toward the formation of unsaturated tetramers (α-C8). Structurally, both catalysts demonstrated thermal stability up to 70 °C, with FC showing significantly greater stability than CC under identical conditions. Additionally, polymerization parameters influenced both catalytic activity and oligomer distribution. The superior performance of FC may be attributed to the configuration of its aryl rings, where half-open or fully half-open windows between the backbones enhance thermal stability and facilitate a more effective cooperative effect between the metal centers. To further improve catalyst performance, the use of a more rigid linker and the incorporation of electron-withdrawing substituents on the spacer have been suggested. Moreover, the synthetic strategy used for these complexes may be extended to the preparation of di- and multinuclear complexes, especially in cases where traditional methods are not suitable. From a computational perspective, recent advancements in modeling the structures of MAO and MMAO cocatalysts allow for more realistic representations of active centers, including the presence of counter anions (i.e., the cocatalyst), thereby providing deeper insights into catalyst behavior and performance.

## Figures and Tables

**Table 1:** Ethylene oligomerization using FC and CC in the presence of MMAO ^a^.

Entry	Cat.	[Al]/[Co]	T (°C)	P (bar)	Yield (g)	Activity ^b^	Distribution of Oligomer ^c^ (%)				
							C_4_	C_6_	C_8_	α-C_8_/ƩC_8_	α-C_8_/ƩC_n_
1	FC	1500	25	2	1.61	8.05	0.0	0.3	99.1	96.4	90.6
2	FC	1500	25	4	1.89	9.45	0.0	0.0	98.9	96.6	91.0
3	CC	1500	25	2	1.03	5.15	0.0	2.3	96.2	95.8	93.9
4	CC	1500	25	4	1.75	8.75	<1	1.5	95.0	96.1	94.4

^a^ Polymerization condition: 80 mL toluene, 20 min, [M] = 6 μmol. ^b^ Activities are expressed as 10^5^ g mol^−1^ [M] h^−1^ ± 3.0 × 10^3^ and determined by GC–MS analysis (based on the fraction of oligomers to the whole oligomerization solution). ^c^ Determined by GC–MS analysis, STD= ±0.04.

**Table 2:** Ethylene oligomerization using FC and CC in the presence of MMAO ^a^.

Entry	Cat.	[Al]/[Co]	T (°C)	P (bar)	Yield (g)	Activity ^b^	Distribution of Oligomer ^c^ (%)				
							C_4_	C_6_	C_8_	α-C_8_/ƩC_8_	α-C_8_/ƩC_n_
5	CC	1500	50	2	0.95	4.75	3.5	3.1	93.1	96.1	98.7
6	CC	1500	75	2	0.67	3.35	6.1	2.2	91.2	96.3	>99
7	FC	1500	50	2	1.49	7.45	1.2	1.1	97.5	97.2	98.4
8	FC	1500	75	2	1.15	5.75	2.2	1.0	96.7	97.3	>99
9	FC	2500	25	2	1.57	7.85	1.1	<1	98.3	95.2	89.3
10	FC	3500	25	2	0.71	3.55	1.9	1.0	96.8	94.8	88.9
11	FC	1500	25	1	0.99	4.95	0.0	<1	>99	96.3	91.8

^a^ Polymerization conditions: 80 mL toluene, 20 min, [M] = 6 μmol. ^b^ Activities are expressed as 10^5^ g mol^−1^ [M] h^−1^ and determined by GC–MS analysis (based on the fraction of oligomers to the whole oligomerization solution). ^c^ Determined by GC–MS analysis.

## Data Availability

The original contributions presented in this study are included in the article/Supplementary Material. Further inquiries can be directed to the corresponding authors.

## Supplementary Materials

The following supporting information can be downloaded at: https://www.mdpi.com/article/10.3390/ma18092123/s1. Figure S1. FT-IR spectrum of FC.; Figure S2. CHNS elemental analysis of FC.; Figure S3. 1H NMR spectrum of FC (after THF and H2O suppressions and baseline correction).; Figure S4. MALDI-TOF spectrum of FC.; Figure S5. FT-IR spectrum of CC.; Figure S6. CHNS elemental analysis of CC.; Figure S7. 1H NMR spectrum of CC (after THF and H2O suppressions and baseline correction).; Figure S8. MALDI-TOF spectrum of CC.; Figure S9. The HOMO and LUMO diagrams of CC and FC.
