# Supplementary material for: Dinuclear Macrocyclic Bis(iminopyridyl) Co- and Fe-Based Catalysts for Ethylene Oligomerization
Source: Materials (Basel). 2025 May 5;18(9):2123. doi: 10.3390/ma18092123 (PMC12073060; doi:10.3390/ma18092123)
Supplement: Supplementary file 1 [file materials-18-02123-s001.zip › materials-3539947-supplementary.pdf]

Supporting information

Dinuclear macrocyclic bis(iminopyridyl) Co- and Fe- based catalysts for ethylene oligomerization

[1] Graduate School of Advanced Science and Technology, Japan Advanced Institute of Science and Technology, 1–1 Asahidai, Nomi, Ishikawa 923–1292, Japan; mostafa@jaist.ac.jp
[2] Key Laboratory of Engineering Plastics and Beijing National Laboratory for Molecular Science, Institute of Chemistry Chinese Academy of Sciences, Beijing 100190, China; myanping@iccas.ac.cn
[3] CAS Research/Education Center for Excellence in Molecular Sciences, University of Chinese Academy of Sciences, Beijing 100049, China
[4] State Key Laboratory for Oxo Synthesis and Selective Oxidation, Lanzhou Institute of Chemical Physics Chinese Academy of Sciences, Lanzhou 730000, China
* Correspondence: whsun@iccas.ac.cn;Tel.: +86 (1062557955)

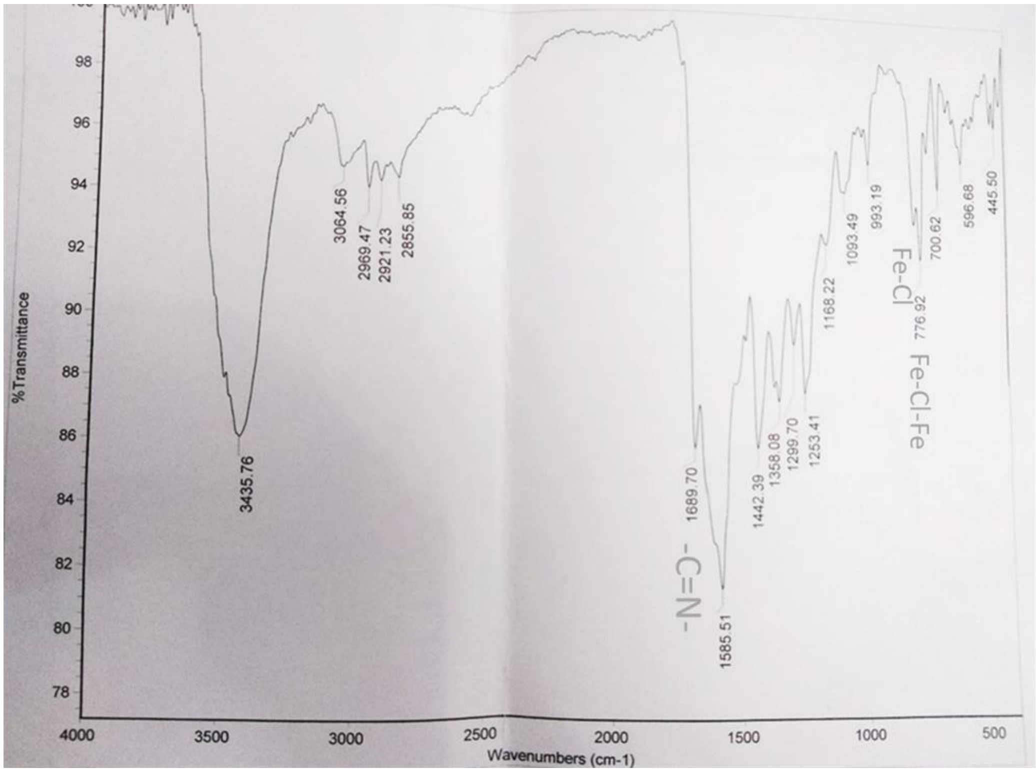

Figure S1. FT-IR spectrum of FC

```
Eager 300 Summarize Results
    Method Name : NCHS
Method Filename : Copy of Copy of N C H S-bkp .mth

       8       1 Sample(s) in Group No : 12
Component Name Average
-------------- --------------
Nitrogen%       10.084591658
Carbon%         51.83179816
Hydrogen%       3.727678101
Sulphur%        0
```

Figure S2. CHNS elemental analysis of FC.

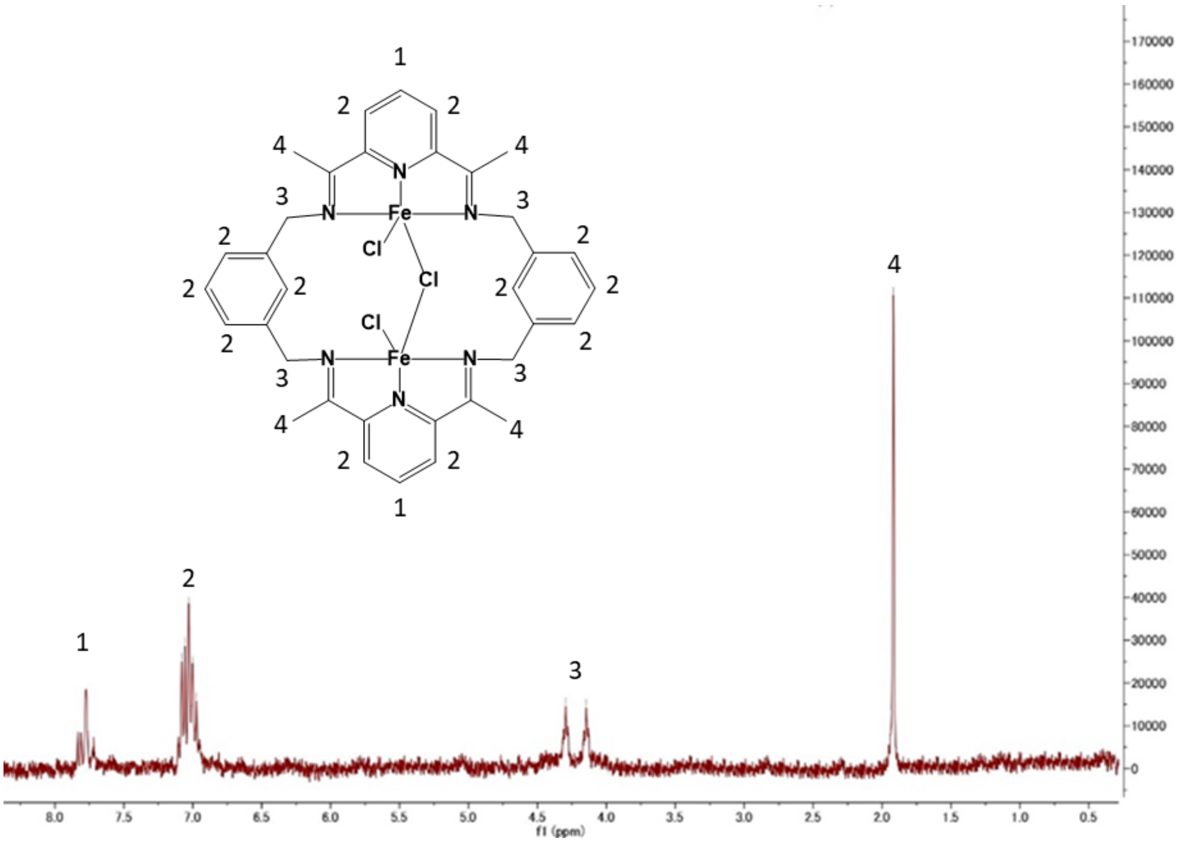

Figure S3. ^{1}H NMR spectrum of FC (after THF and H_2O suppressions and baseline correction).

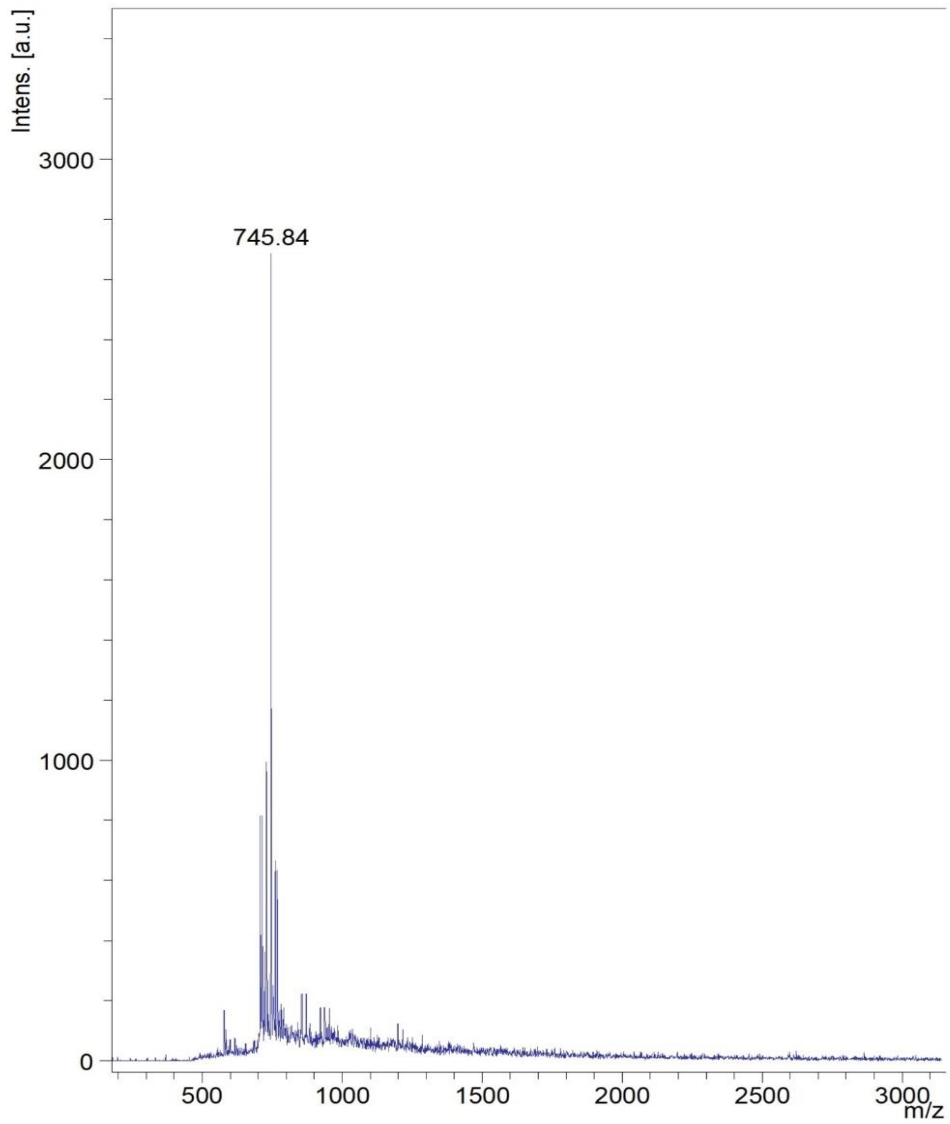

Figure S4. MALDI-TOF spectrum of FC.

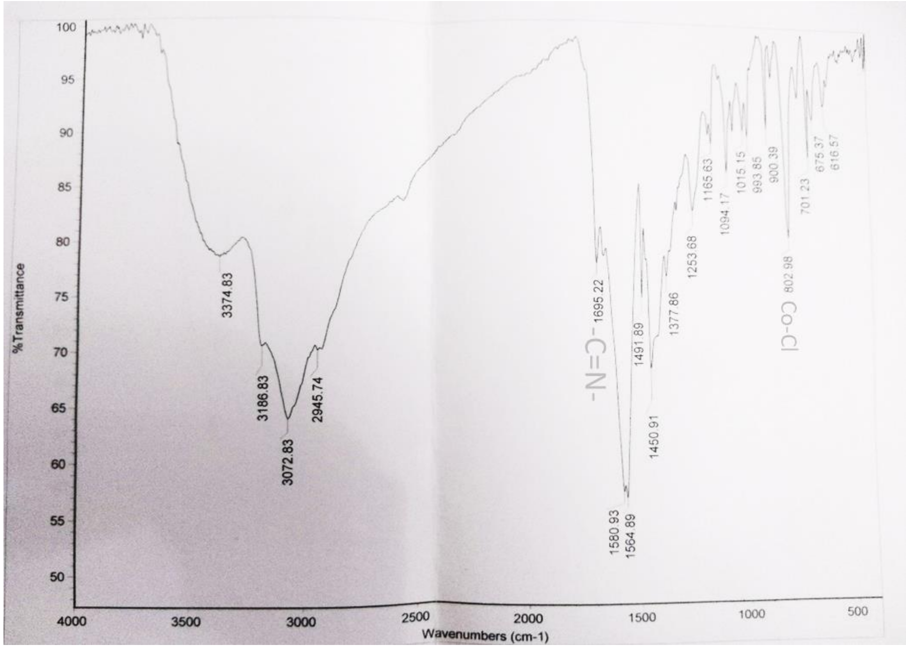

Figure S5. FT-IR spectrum of CC.

```
Eager 300 Summarize Results
    Method Name : NCHS
Method Filename : Copy of Copy of N C H S-bkp .mth

       8       1 Sample(s) in Group No : 11
Component Name Average
-------------- --------------
Nitrogen%       9.872901485
Carbon%         51.20104611
Hydrogen%       3.717677601
Sulphur%        0
```

Figure S6. CHNS elemental analysis of CC.

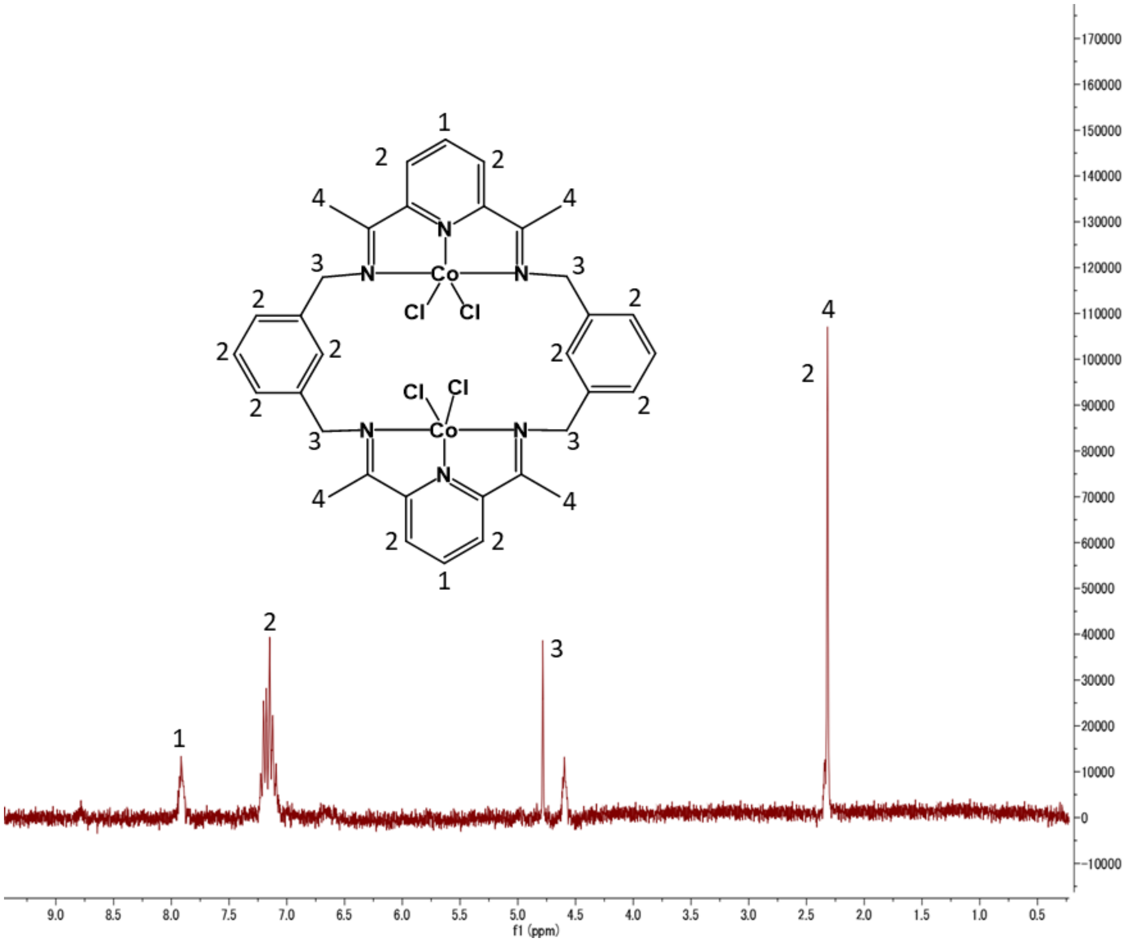

Figure S7. ^{1}H NMR spectrum of CC (after THF and H_2O suppressions and baseline correction).

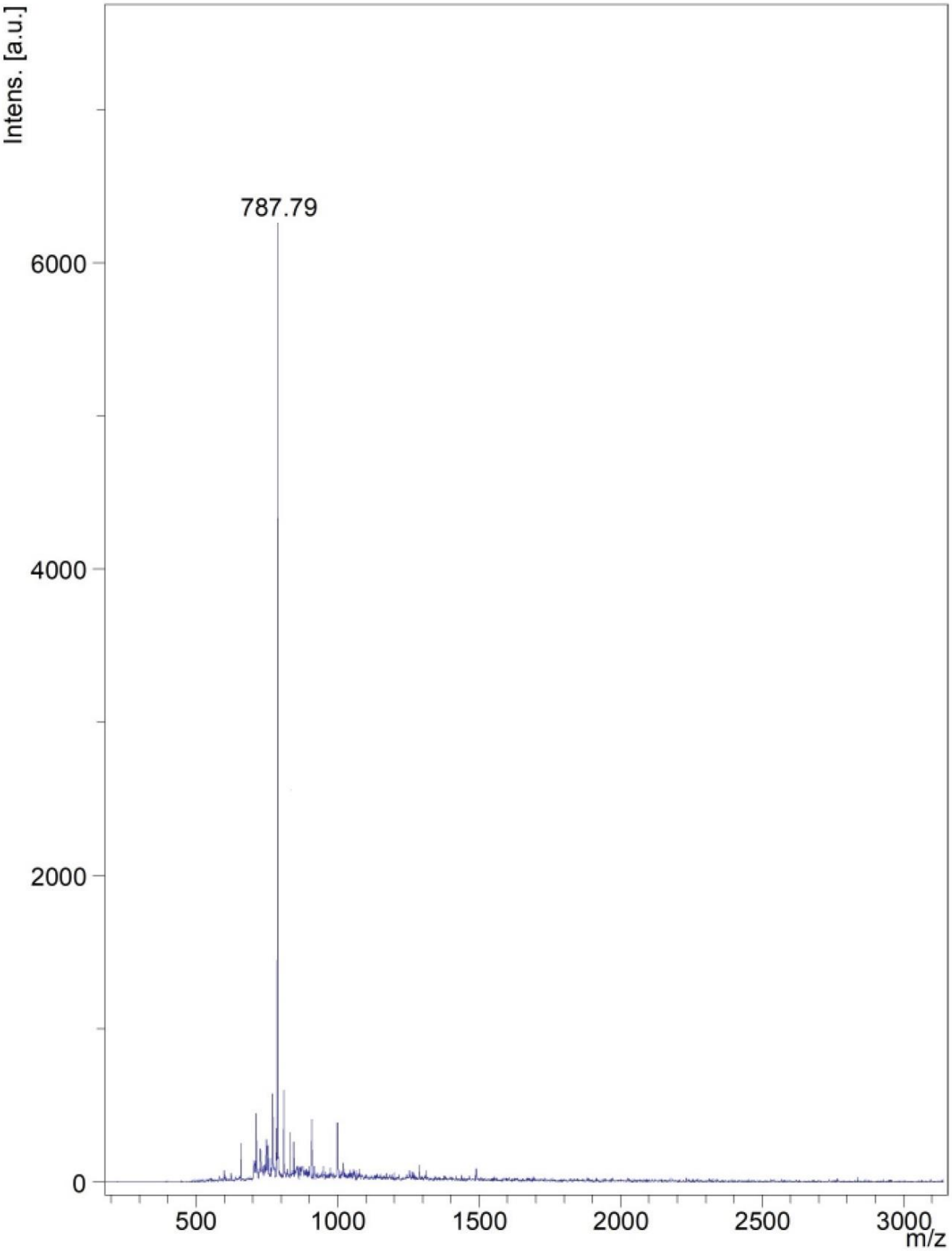

Figure S8. MALDI-TOF spectrum of CC.

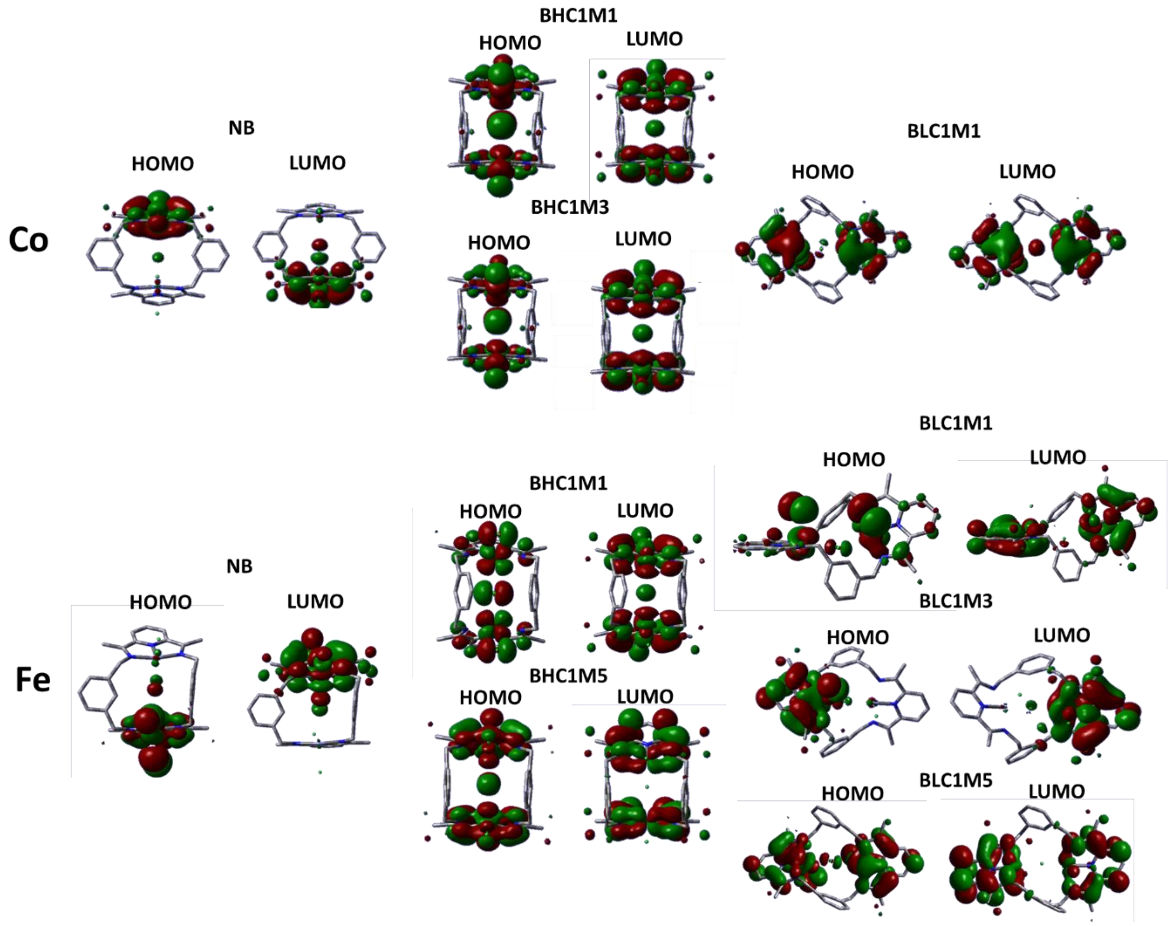

Figure S9. The HOMO and LUMO diagrams of CC and FC.
